# The relationship between life satisfaction, proactivity, perceived stress and work engagement in educationalists working in alternative settings in England

**DOI:** 10.1186/s40359-025-03734-5

**Published:** 2025-11-27

**Authors:** Ceri Rowley, Boban Simonovic, Katia C. Vione

**Affiliations:** 1 School of Psychology, University of Derby, Kedleston Road, Derby, DE22 1GB UK; 2 School of Psychology, University of Birmingham, Birmingham, UK

**Keywords:** Work engagement, Life satisfaction, Proactive personality, Alternative education

## Abstract

Although work engagement is recognised as a key state influencing staff well-being and performance, educational research has often prioritised organisational factors, overlooking individual-level variables. This study addresses this gap by examining individual characteristics among staff working in Alternative Provision settings (APs), such as Pupil Referral Units (PRUs) and hospital education services across England. This research investigated the relationships between life satisfaction (SWL), perceived stress (PS), proactive personality (PPS), and work engagement (Vigour, Dedication, Absorption). Proactive personality was conceptualised as a relatively stable personal trait, whereas life satisfaction, perceived stress, and work engagement were treated as context-dependent states or attitudes. A total of 101 current teachers and support staff were recruited with assistance from the National Association of Hospital Education and the PRUs Organisation (PRUsAP). Data were collected using validated scales for each construct, and multiple regression and moderation analyses were conducted. Key findings revealed that life satisfaction and proactive personality were significant positive predictors of all three dimensions of work engagement. Conversely, perceived stress negatively predicted vigour and dedication. Importantly, proactive personality moderated the relationship between life satisfaction and vigour: for educators with lower proactivity, higher life satisfaction was strongly associated with increased vigour, an effect that diminished as proactive personality increased. The full models accounted for substantial variance—57% in vigour, 31% in dedication, and 40% in absorption. These findings suggest that interventions aimed at enhancing life satisfaction may be especially beneficial for staff less naturally inclined towards proactive behaviour. The results highlight the value of recognising individual differences in promoting well-being and engagement in alternative educational contexts.

## Introduction

Alternative provision (AP) in education plays a crucial role in supporting students experiencing behavioural difficulties, chronic illness, or other challenges such as neurodevelopmental disorders and mental health conditions that impede their participation in mainstream education; these are often referred to as additional learning barriers, encompassing a wide range of cognitive, emotional, and physical factors [1]. AP in England includes Pupil Referral Units (PRUs), hospital schooling, and off-site placements for students unable to attend mainstream settings,these forms have recently been mapped and critiqued within the UK context [2, 3]. In this context, teacher work engagement is of vital importance, as empirical evidence demonstrates that students frequently emulate the engagement behaviours exhibited by their educators [4]. The Department for Education has reported significant levels of teacher attrition, with 32.5% of newly qualified teachers leaving the profession within five years of qualification and 42% leaving within their first decade [2]. While the trend has remained consistently high over recent years rather than sharply increasing, this sustained attrition remains a serious concern for workforce stability [5]. Within AP specifically, staff turnover rates are notably higher than in mainstream settings, with some AP schools reporting annual turnover rates exceeding 25% [6]. Given reports of rising school exclusions and shifts toward greater use of AP in recent years (e.g., [7]), pressure on AP settings may intensify further. High attrition in AP settings disrupts care continuity, reduces specialist expertise, strains resources, and diminishes student engagement and stability, which are vital for supporting vulnerable learners [1, 4]. These issues are compounded by systemic challenges such as low life satisfaction among educators, resource constraints, and limited policy influence [2, 8, 9]. Understanding the drivers of work engagement is therefore essential to informing organisational support strategies, reducing staff turnover, and improving outcomes for students in AP [10].

Work engagement remains a complex but increasingly important construct in education due to its dynamic nature and practical relevance [8]. Work engagement reflects a sustainable, balanced investment of physical, cognitive, and emotional resources, which is essential for the demanding, relational work of teaching, particularly in AP settings [7, 11, 12]. Foundational theories characterise engagement as a multifaceted expression of self—physically, emotionally, and cognitively—moderated by meaningfulness, safety, and resource availability [13, 14]. Building on this, work engagement is defined as a positive state featuring vigour, dedication, and absorption, influenced by both energetic and motivational processes, where organisational job demands predict burnout and resources foster engagement [15, 16]. Crucially, individual factors such as personal resources—including traits seen as a subset of these resources—and well-being play a vital role [12, 17]. This integrative perspective aligns with evidence that, despite occupational stress, teachers often derive fulfilment from their roles [18], Ofsted, 2023). Nonetheless, further research is needed to clarify the individual characteristics driving engagement, especially personal traits and resources, and their interplay—largely independent of organisational factors, which the literature has arguably over‑emphasised [7, 19, 20]—highlighting the continuing evolution of work engagement as a multidimensional, context-dependent construct.

### Theoretical framework

The Job Demands-Resources (JD-R) model, initially proposed by Demerouti et al. [21], asserts that job resources positively influence work engagement and performance, encouraging organisations to facilitate job crafting and resource utilisation [22]. As the model evolved, it incorporated personal resources—such as self-efficacy, optimism, and resilience—emphasising individuals’ confidence in shaping their work environments alongside traditional organisational factors like autonomy, feedback, and social support [12, 17]. This dual focus is particularly pertinent to AP education, where organisational resources are often scarce and unpredictable (Ofsted, 2021 [23],). In this context, personal resources and work motivation enable teachers to manage professional challenges effectively [24], sustaining the core engagement dimensions of vigour, dedication, and absorption measured by the Utrecht Work Engagement Scale [16]. These aspects are critical for educators working with vulnerable students, underpinning consistency, emotional availability, and pedagogical creativity essential for positive outcomes [25, 26]. For example, when teachers experience high vigour, they possess the physical and mental stamina to navigate challenging behaviours,dedication ensures sustained commitment despite setbacks; and absorption facilitates the deep focus required for differentiated instruction and relationship building with students who have complex needs [27].

Although work engagement research has traditionally emphasised organisational variables, evidence suggests that external influences such as personal well-being warrant greater attention [28]. Notably, teacher engagement remains under-researched despite its documented association with improved student outcomes and retention- a gap that is critical in AP settings where demands are high and resources limited [29]. Recent reviews have begun to address these gaps, with Ofsted [30] highlighting the predictive relationship between teacher engagement and student engagement, which subsequently impacts academic achievement, while Owen et al. [31] identify personal resources as critical yet underexplored factors influencing teacher well-being and engagement. However, these reviews also reveal that the AP sector remains virtually absent from the teacher engagement literature, with research concentrated predominantly in mainstream educational settings [30]. This represents a critical omission, as the unique challenges of AP—including higher staff turnover, more complex student needs, and more limited resources—may fundamentally alter the predictors and processes underlying work engagement. This gap is particularly significant given the potential consequences for educational outcomes and teacher retention in these vulnerable educational contexts.

### Life satisfaction as a personal resource for work engagement

Research exploring the relationship between life satisfaction and work engagement has largely considered engagement as a precursor rather than a consequence of life satisfaction, leaving the predictive role of life satisfaction on engagement relatively under-examined [28, 32, 33]. Theoretically, life satisfaction constitutes a vital personal resource that may enhance work engagement through multiple pathways. According to Hobfoll’s [34] Conservation of Resources (COR) theory, individuals with higher life satisfaction possess an enriched pool of psychological resources, enabling sustained investment in their professional roles. Complementing this, Fredrickson’s [35] broaden-and-build theory proposes that the positive emotions linked to life satisfaction expand cognitive and behavioural repertoires, fostering the development of enduring psychological resources that support core engagement dimensions such as vigour, dedication, and absorption. This is especially resonant in AP teaching contexts, where educators confront chronic stressors and limited organisational support [1, 23, 36], here, life satisfaction may buffer against resource depletion, sustaining engagement under adversity. Empirical support for this theoretical positioning aligns with Ferreira et al.’s [28] findings that work engagement is influenced by life satisfaction in addition to work-related demands and resources, reinforcing the importance of considering well-being beyond workplace factors. Measurement choice is also important: the Satisfaction with Life Scale (SWL) is widely acknowledged as an effective tool for capturing the cognitive dimension of well-being, distinguishing it from affective components, thereby providing a stable indicator of life satisfaction relevant for predicting engagement over time [37–39]. While Çelik [40] explores associations between life satisfaction and work behaviours, the idea of a reciprocal relationship—where life satisfaction promotes proactive behaviours that in turn sustain and amplify engagement—is a synthesis derived from the wider literature rather than an explicit claim made by Çelik. This conceptualisation is thus discussed in more detail later within the JD-R framework, where it aligns more closely with the study’s focus and findings. Although recent research has increasingly examined life satisfaction as an antecedent of work engagement, significant gaps remain, especially concerning potential moderating factors and context-specific investigations.

Eldor et al. [41] assert that life satisfaction can foster a positive outlook and personal resource development, potentially leading to increased work engagement through a desire for personal growth. Similarly, longitudinal evidence shows a bidirectional relationship between life satisfaction and work/study engagement over eight years [42], whereby, life satisfaction predicted higher engagement, which subsequently enhanced life satisfaction as individuals progressed through educational and career stages [43]. This relationship has been observed across various educational settings. Žnidaršič and Marič [44] found a positive correlation between life satisfaction and work engagement in higher education staff, while De Stasio et al. [32] reported that subjective well-being influenced work engagement among Italian kindergarten teachers. However, these studies have exclusively examined mainstream or higher education contexts, and none have investigated whether stable personality characteristics might moderate the life satisfaction-engagement relationship. Furthermore, AP settings—characterised by higher stress, greater student complexity, and fewer organisational resources than mainstream schools [1, 23, 36, 45]—may represent a context where the interplay between life satisfaction and personality traits is particularly consequential yet remains entirely unexplored. Given Ofsted's [36] report of lower life satisfaction among educationalists compared to the general public, further research into this relationship in underserved educational contexts and examining potential personality-based moderators could provide crucial insights for supporting educators' well-being and effectiveness in their roles.

### Perceived stress and work engagement

Concurrent with life satisfaction, perceived stress exerts a complex influence on work engagement, informed by both theoretical and empirical evidence. The COR theory contextualises stress as the perception or reality of resource threat or loss, with resource availability—and personal characteristics—modulating stress impact [34]. Casper et al. [46] and Keech et al. [47, 48] advance the stress mindset framework, recognising that individual beliefs about stress can transform its physiological and behavioural consequences, rendering stress either debilitating or facilitative. Measuring perceived stress through the Perceived Stress Scale (PS) offers a comprehensive view of individuals’ sense of control, predictability, and overwhelm, unconfined to specific situations [49]. In education, stress spans personal and professional domains and poses significant challenges to teachers’ well-being and efficacy [50, 51]. Although stress often correlates negatively with work engagement and student outcomes [52], positive engagement amidst stress has been observed, particularly where educators have developed coping strategies that safeguard their resource reservoirs [53–55]. These nuanced findings highlight the intricate interplay between life satisfaction, perceived stress, and work engagement, emphasising the empirical and practical value of further research elucidating these dynamics within the uniquely demanding setting of AP education.

### Proactive personality: a trait-based resource for engagement

Beyond well-being factors such as life satisfaction and perceived stress, individual personality characteristics may also serve as critical personal resources for sustaining work engagement, especially in challenging AP settings where organisational support is often limited. Proactive personality—a stable dispositional trait reflecting an individual’s inclination to take initiative and influence their environment [56]—is particularly relevant in this context. Research across stressful professions, including teaching and healthcare, consistently shows that controllable factors, such as proactive behaviours, are primary predictors of engagement and well-being [53, 55]. This aligns with Bakker et al.’s [57] Job Demands-Resources (JD-R) model proposition that individuals actively seek to shape their environment through proactive behaviours. While proactivity is distinct from innovation, it entails deliberate, self-initiated attempts to improve current circumstances rather than passively accepting them [58, 59]. For this study, proactive personality is conceptualised as a stable trait assessed by the Proactive Personality Scale, distinguishing it from context-specific proactive behaviours like job crafting or voice, which may result from the trait [60]. By focusing on proactive personality as a personal resource within the JD-R framework, we examine whether this trait enables teachers to generate engagement resources independently of external job demands and organisational constraints. Prior studies suggest proactive personality positively predicts work engagement, with evidence also highlighting a bidirectional relationship between proactive behaviour and engagement [61, 62]. This highlights the complex interplay between enduring traits, dynamic behaviours, and engagement outcomes, warranting nuanced understanding in fostering engagement in high-demand professional environments.

Within the JD-R framework, proactive personality functions as a personal resource rather than a demand [17, 19]. Personal resources are defined as positive self-evaluations linked to resilience and individuals' sense of their ability to control and impact their environment successfully. Proactive personality aligns with this definition as it represents individuals' stable tendency to enact environmental change and create opportunities, thereby enabling them to generate additional job resources (such as autonomy, feedback, and social support) even in constrained contexts [12] [63]. This resource-generating capacity is particularly salient in alternative provision settings, where organisational resources are often limited, making teachers' ability to proactively create their own resources a crucial determinant of sustained engagement. Importantly, research by Jorg [64] highlights that while professional competences like self-efficacy predict teachers’ concurrent proactive behaviour, job design characteristics such as time pressure can moderate this effect, with proactive behaviour subsequently enhancing teachers’ self-efficacy and perceptions of job demands over time. This longitudinal perspective underscores the dynamic and reciprocal relationships involved in JD-R theory more broadly, indicating that proactive personality and behaviour not only respond to but also shape work conditions, facilitating sustained engagement amid challenging educational settings. However, the current study focuses specifically on the unidirectional influence of proactive personality as a resource leading to engagement in alternative provision educators. Thus, proactive personality operates as a personal resource that facilitates engagement by enabling individuals to actively construct favourable work conditions rather than passively responding to existing demands [65, 66].

### An integrated model of individual resources and work engagement in alternative provision

The foregoing review establishes that work engagement in AP teaching is influenced by both stable individual characteristics and broader well-being factors. To develop a comprehensive understanding of teacher work engagement in these challenging settings, we adopt an integrative framework examining three key individual-level predictors: life satisfaction (a cognitive evaluation of overall well-being), perceived stress (an assessment of one's coping capacity), and proactive personality (a stable trait reflecting the tendency to enact environmental change). These variables were selected because they represent conceptually distinct yet complementary facets of individual differences that are theoretically linked to the personal resources required for sustained engagement in high-demand, low-resource educational contexts. Life satisfaction reflects the overall psychological capital available for work investment; perceived stress captures individuals' beliefs about their capacity to manage demands; and proactive personality represents the dispositional drive to actively shape one's environment—a particularly relevant trait in AP where teachers must often create their own resources and solutions in the absence of systemic support [1, 23],Ofsted, 2021, [30]). Importantly, whilst life satisfaction and stress represent potentially malleable states that organisations might influence through support interventions, proactive personality represents a more stable individual difference that may determine how teachers utilise their well-being resources. This distinction is crucial for understanding not only whether these factors predict engagement, but also how they interact to shape engagement in educationalists working in alternative settings.

### Hypotheses development

This study aims to provide a more comprehensive understanding of teacher work engagement in AP settings by simultaneously investigating the roles of distinct facets of individual well-being (life satisfaction and perceived stress) and a key personality trait (proactive personality). While previous research has examined these constructs individually in relation to work engagement, and whilst the main effects of life satisfaction, perceived stress, and proactive personality on engagement are theoretically well established and empirically supported (as reviewed above), three critical gaps remain. First, these factors have rarely been examined simultaneously in a single model, leaving uncertain their relative contributions and potential interdependencies. Second, the AP context—characterised by unique challenges including vulnerable student populations, limited resources, high staff turnover, and systemic constraints [7, 30, 45]—remains severely understudied, despite representing a critical educational sector where understanding engagement determinants is particularly urgent. Third, and most importantly, theoretical ambiguity persists regarding whether personal well-being resources (such as life satisfaction) uniformly benefit all individuals, or whether their impact depends upon stable personality characteristics.

Accordingly, while we test the expected main effects of life satisfaction, perceived stress, and proactive personality (H1-H3) to establish whether established patterns replicate in this underserved context, our primary theoretical contribution centres on the proposed moderation effect (H4). Specifically, we propose that while life satisfaction and perceived stress will directly influence different dimensions of work engagement, the impact of life satisfaction will not be uniform across all individuals. Instead, we posit that an individual's proactive personality will critically moderate the relationship between life satisfaction and work engagement. As highly proactive individuals are inherently driven to influence their environment and seek opportunities for growth (e.g., [65, 66]), their work engagement may be less dependent on their general life satisfaction, as they might generate their own resources or find engagement through active problem-solving even in challenging contexts [12]. Conversely, for those with lower proactive tendencies, a higher sense of overall life satisfaction could serve as a more vital personal resource, buffering against disengagement and fostering the necessary vigour, dedication, and absorption for their demanding roles [34, 37]. This moderation hypothesis is theoretically grounded in the JD-R theory's emphasis on personal resource interactions [12] and extends Conservation of Resources theory [34] by suggesting that personality traits determine how individuals mobilise their well-being resources for work investment. Understanding this interaction is particularly crucial in alternative provision settings, where teachers cannot rely upon abundant organisational resources and must instead leverage their personal resources strategically.

This study employs the Utrecht Work Engagement Scale (UWES) and its subscales – Vigour, Dedication, and Absorption – as separate outcome variables to provide a fine-grained analysis of engagement components, aligning with its conceptualisation as a multidimensional construct [16]. Examining these dimensions separately allows us to determine whether the proposed relationships operate uniformly across all facets of engagement or differentially affect teachers' energy levels (vigour), emotional commitment (dedication), and immersion in work (absorption)—a distinction that may inform more targeted interventions to support AP educators.

Based on available research, we propose the following hypotheses: H1: Life satisfaction positively predicts (a) Vigour, (b) Dedication, and (c) Absorption. H2: Perceived stress negatively predicts (a) Vigour, (b) Dedication, and (c) Absorption. H3: Proactive personality positively predicts (a) Vigour, (b) Dedication, and (c) Absorption. H4: Proactive personality will moderate the relationship between life satisfaction and work engagement sub-dimensions, such that the positive effect of life satisfaction on engagement will be stronger when proactive personality is low, and weaker when proactive personality is high for: (a) Vigour, (b) Dedication, and (c) Absorption.

## Method

### Participants

Sample size was estimated based on G*power calculations [67] for a regression analyses, estimating a small to medium effect size, and 80% power. A minimum of 95 participants was required. Inclusion criteria were being over the age of 18 and working in an alternative educational provision in England. Alternative provision comprises educational settings outside mainstream and special schools, designed for students unable to attend traditional environments. It includes pupil referral units (PRUs), alternative provision academies, free schools, hospital schools, and tailored programmes commissioned by local authorities or schools for pupils at risk of exclusion, those who have been excluded, have medical needs, or face other barriers to mainstream attendance [2, 45]). Staff in these settings typically work with smaller class sizes, students with complex behavioural and emotional needs, and often have greater autonomy in curriculum delivery compared to mainstream settings, whilst facing challenges including limited resources, higher staff turnover, and systemic under-recognition [1, 23, 30]. Participants were required to be involved in a student’s learning, and therefore teaching assistants, teachers, and senior leaders with involvement in teaching, learning and behaviour were invited to take part. In addition to being over 18, due to the nature of the surveys, participants were excluded if they had taken time off work with a stress-related illness in the last academic year. A total of 121 respondents took part in the study. Of those, nine were incomplete, six did not meet the inclusion criteria, and five did not give consent both at the start and the end of the survey. Therefore, analysis includes responses from 101 participants in total. No analysis of demographic information was included in the design of the study, and therefore, in line with General Data Protection Regulation (GDPR) guidance on personal data [68] this information was not collected.

### Materials

#### Satisfaction With Life (SWL [37]***;***)

Participants responded to the five statements of the SWL. For example, ‘I am satisfied with my life’. Responses were recorded on a 7-point Likert-type scale from 1 (strongly disagree) to 7 (strongly agree). Judgements are based on personal view of how satisfied they are with what they have, compared with the standard they set for themselves [37]. Pavot and Diener’s [38] review of the scale demonstrated good internal consistency reporting Cronbach alpha estimates between α = 0.79–0.89.79.89, while in the present study it was calculated as α = 0.81.

#### Perceived Stress Scale (PS: [69])

This was measured using the PS, which comprises ten questions measured on a 5-point Likert-type scale from 0 (never) to 4 (very often). Questions measure how often their experiences lead them to feel stressed using questions such as ‘in the last month, how often have you felt nervous and “stressed”?’ Cohen and Janicki‐Deverts, [70] report studies with internal reliabilities of α = 0.78 and α = 0.91. In the present study, Cronbach’s alpha was calculated at α = 0.86.

#### Proactivity Personality Scale (PPS; [56])

Participants completed the PPS, consisting of 17 statements such as ‘if I see something I don’t like, I fix it’. Responses are recorded on a 7-point Likert-type scale from 1 (strongly disagree) to 7 (strongly agree). Bateman and Crant [56] demonstrated reliability estimate of α = 0.89, 0.87 and 0.87 for the scale. In the present study, Cronbach’s alpha was calculated as α = 0.92.

#### Work Engagement (UWES; [16])

The 17-items UWES was used to measure participants mentality at work, for example ‘at my work I am bursting with energy’. Responses are recorded on a 7-point Likert-type scale from 0 (never) to 6 (every day or always). The scale can be used as a complete scale, or separated into three sub-components of Vigour, Dedication and Absorption [16]. Internal consistency for the UWES subscales was reported as good in the past by Field and Buitendach [33] (e.g., α = 0.78. α = 0.88 and α = 0.91). In the present study, the complete UWES 17-item scale Cronbach’s alpha was calculated at α = 0.89. The Cronbach’s alpha was also calculated separately for subscales (Vigour’s α = 0.81,Dedication’s α = 0.88 and Absorption’s α = 0.75).

### Procedure

After receiving ethical approval for the study, Twitter was used for recruitment, with support from the National Association of Hospital Education (NAHE) and the National Organisation of Professionals concerned with Provision and Outcomes for Students at Risk of Educational or Social Exclusion (PRUsAP), who shared the invitation to participate each time the researcher posted it on their personal account. From here, the survey was shared several times by other users, including by another organisation, The AP Research Network. Whilst social media recruitment introduces limitations including potential self-selection bias and difficulty verifying respondent credentials, several safeguards were implemented to ensure sample integrity. First, the survey included specific screening questions requiring participants to confirm their current employment in an alternative provision setting in England and their role involving direct student teaching, learning, or behaviour support. Second, participants were required to demonstrate familiarity with alternative provision terminology and working conditions through these screening items. Third, the survey was shared exclusively through professional networks and organisations specifically serving alternative provision educators (NAHE, PRUsAP, and the AP Research Network), making it unlikely that individuals outside this professional community would encounter or complete the survey. Fourth, responses were manually reviewed for consistency and plausibility, with six responses excluded for not meeting inclusion criteria. Whilst these measures cannot guarantee absolute verification of respondent identity—a limitation we acknowledge—they substantially reduce the likelihood of invalid responses. Furthermore, social media recruitment via professional networks has been successfully employed in previous teacher well-being research (e.g., [71, 72]) and offers advantages including access to geographically dispersed alternative provision staff who are otherwise difficult to reach through traditional recruitment methods.

Qualtrics was used to inform participants about the study and to collect answers to four questionnaires. Participants were first given a brief introduction to the research and informed of the anonymity and confidentiality of their answers as well as their right to withdraw and how their data would be used. Participants provided written informed consent in accordance with approved university research protocols, the British Psychological Society ethical guidelines, the Data Protection Act (1998), and the GDPR (2018). They first confirmed their suitability to complete the study, followed by the completion of four questionnaires in a randomised order. At the end, a debrief reminded the participants of the ethical approach to the study, their rights, and asked them to reconfirm they were still happy to participate. They were also given information relating to organisations that could help and support them should it be required. This process took approximately 20 min to complete.

### Analytic strategy and scoring

Prior to analysis, data were subjected to both graphical and statistical screening. To address the non-normal distribution observed across variables, a logarithmic transformation was applied to the data and used in all subsequent analyses. Variables were scored according to their respective scoring guidelines. A total score was computed for each variable and subscale, and data were examined to ensure accuracy of entry. Inter-correlations between all variables were first examined to assess the bivariate relationships between predictors and outcomes, verify that the three work engagement subscales (Vigour, Dedication, and Absorption) were sufficiently distinct to warrant separate analysis (rather than relying on only a total engagement score), check for potential multicollinearity issues among predictors, and provide preliminary evidence regarding the expected directions of relationships specified in our hypotheses. This correlational analysis serves as a foundational step before proceeding to more complex regression and moderation analyses. We then conducted three separate multiple regressions with SWL, PS, and PPS as predictors of work engagement's subscales: Vigour, Dedication, and Absorption. Lastly, three separate bootstrapped moderation analyses were conducted to examine whether the relationship between SWL and work engagement's subscales was moderated by PPS, while controlling for potential covariates of PS. PS was included as a covariate in the moderation analyses for two theoretical reasons. First, perceived stress and life satisfaction are conceptually related constructs (both reflecting aspects of personal well-being) and were moderately correlated in our data, suggesting they share variance. Controlling for PS allows us to isolate the unique effect of life satisfaction on work engagement and determine whether the moderation effect of proactive personality on the life satisfaction-engagement relationship holds above and beyond the influence of stress perceptions. Second, our theoretical framework (Conservation of Resources theory; [34]) suggests that both life satisfaction and perceived stress function as personal resources (or resource depletion indicators) that may influence work engagement. By controlling for PS, we can determine whether the hypothesised moderation effect is specific to the life satisfaction pathway or alternatively might be explained by general well-being or stress-related processes. We used the Process macro for SPSS [73], with 5,000 bootstrapping re-samples and bias-corrected 95% Confidence Intervals (CIs) to test the moderation effects. IBM SPSS Statistics 27 software package for Windows was used to analyse data.

## Results

### Inter-correlations between all dependent measures

Table 1 presents inter-correlations between all dependent measures.

**Table 1:** Inter-correlations, descriptive statistics, and reliabilities for all Variables (*N* = 101)

Variables	M	SD	α	1	2	3	4	5	6
1. PS	2.01	0.58	.86	-					
2. SWL	4.82	1.23	.81	-.547*	-				
3. PPS	5.42	0.87	.92	-.152	.115	-			
4. Vigour	4.21	1.02	.81	-.375*	.337*	.693*	-		
5. Dedication	5.03	0.95	.88	-.346*	.294**	.464*	.760*	-	
6. Absorption	4.45	0.89	.75	-.188	.262**	.605*	.830*	.732*	

Please note all *significant at *p* <.001; all ** significant at *p* <.01

As expected, the three dimensions of work engagement (Vigour, Dedication, Absorption) are highly inter-correlated, reinforcing their conceptual unity whilst demonstrating sufficient distinctiveness (r values ranging from 0.732 to 0.830) to warrant separate examination. All scales demonstrated acceptable to excellent internal consistency (α ranging from 0.75 to 0.92). SWL showed moderate, significant positive correlations with all three dimensions of work engagement, indicating that individuals with higher life satisfaction tend to be more engaged at work. PPS showed strong to moderate-strong, significant positive correlations with all three dimensions of work engagement, highlighting its importance as an individual resource for engagement. PS was negatively and significantly correlated with Vigour and Dedication, but not with Absorption. This suggests that stress primarily impacts the energetic and committed aspects of engagement, but less so the sense of being engrossed in one's work.

### Multiple regressions and moderation analyses

#### Hypothesis testing: main effects (H1-H3)

The first multiple regression (Enter method) demonstrated that the model incorporating PS, SWL and PPS, significantly predicted Vigour, explaining 56% of its variance. (R^2^ = 0.57; R^2^_adjust_ = 0.56; (*F*(3,97) = 43.21, *p* < 0.001). The Beta for SWL and PPS showed a positive correlation, and the Beta for PS showed a negative correlation. This indicated that higher life satisfaction (SWL) and proactive personality (PPS) and lower perceived stress (PS) were associated with higher Vigour (Table 2), providing support for H1a, H2a, and H3a.

**Table 2:** Multiple Regression Analysis of Satisfaction with Life, Proactive Personality and Stress Perception as Predictors of Vigour (Regression 1), Dedication (Regression 2) and Absorption (Regression 3) work engagement

Outcome Variable	Predictor	β	SE	95% CI	t	p
**Vigour**						
(R^2^ =.57, *F*(3,97) = 43.21, *p* <.001)	SWL	.11	.05	[.01,.21]	2.11	.04*
	PS	-.15	.06	[-.27, -.02]	−2.33	.02*
	PPS	.63	.06	[.50,.76]	9.82	<.001*
**Dedication**						
(R^2^ =.31, *F*(3,97) = 14.20, *p* <.001)	SWL	.09	.07	[-.05,.23]	1.29	.20
	PS	-.17	.08	[-.33, -.01]	−2.08	.04*
	PPS	.42	.09	[.25,.60]	4.89	<.001*
**Absorption**						
(R^2^ =.40, *F*(3,97) = 21.84, *p* <.001)	SWL	.12	.05	[.01,.22]	2.17	.03*
	PS	.01	.06	[-.12,.13]	0.13	.90
	PPS	.49	.07	[.36,.62]	7.36	<.001*

All * Are significant; Regression 1: Durbin Watson = 1.85; VIF= 1.43; 1.44; 1.02. Regression 2: Durbin Watson = 1.77; VIF= 1.43; 1.44; 1.02. Regression 3: Durbin Watson = 1.83; VIF= 1.43; 1.44; 1.02

The second multiple regression (Enter method) demonstrated that the model incorporating PS, SWL and PPS, significantly predicted dedication, explaining 28% of the variability in dedication (R^2^ = 0.31; R^2^_adjust_ = 0.28; (*F*(3,97) = 14.20, *p* < 0.001). The Beta for PPS showed a positive correlation, and the Beta for PS showed a negative correlation. This indicated that the higher scores on PPS and lower PS scores were associated with higher Dedication (Table 2). SWL was not a significant predictor. These results provide support for H2b and H3b, but not for H1b.

The third multiple regression (Enter method) demonstrated that the model incorporating PS, SWL and PPS, significantly predicted Absorption, explaining 38% of the variability in Absorption (R^2^ = 0.40; R^2^_adjust_ = 0.38; (*F*(3,100) = 21.84, *p* < 0.001). The Beta for SWL and PPS showed positive correlations. This indicated that the higher score on SWL and PPS were associated with higher Absorption (Table 2). PS was not a significant predictor. These results provide support for H1c and H3c, but not for H2c.

### Hypothesis testing: moderation effects (H4)

To test H4 (whether proactive personality moderates the relationship between life satisfaction and work engagement subscales), we conducted three separate moderation analyses using the PROCESS macro (Model 1; [73]) with 5,000 bootstrap resamples. In each analysis, SWL served as the independent variable, one work engagement subscale (Vigour, Dedication, or Absorption) served as the dependent variable, PPS served as the moderator, and PS was included as a covariate. The critical test of moderation is the interaction term (SWL × PPS).

### Moderation analysis 1: vigour as outcome

The overall moderation model was significant, *F*(4,96) = 35.76, *p* < 0.001, R^2^ = 0.60, accounting for 60% of the variance in Vigour. Critically, the interaction term (SWL × PPS) was statistically significant (β = −1.17, SE = 0.46, t = −2.56, p = 0.01, 95% CI [−2.08, −0.26]), indicating that proactive personality significantly moderates the relationship between life satisfaction and Vigour. This provides support for H4a.

To interpret the nature of this moderation effect, we conducted simple slopes analyses at three levels of PPS: low (1 SD below the mean, value = 1.86), medium (mean, value = 1.94), and high (1 SD above the mean, value = 1.99). Results are presented in Table 3.

**Table 3:** Conditional effects of SWL on vigour at different levels of PPS

PPS	β	SE	t	p	LLCI	ULCI
1.86	.19	.06	3.08	.002*	.06	.30
1.94	.09	.05	1.70	.09	-.01	.19
1.99	−03	.05	.58	.56	-.08	.15

* Significant at p <.01. β represents the effect of SWL on Vigour at each level of PPS

As hypothesised, simple slopes analysis revealed that the positive effect of life satisfaction on Vigour was significant and strongest for participants with low proactive personality (β = 0.19, p = 0.002), non-significant at mean levels of proactive personality (β = 0.09, p = 0.09), and non-significant for those with high proactive personality (β = 0.03, p = 0.56). This pattern supports H4a: for individuals low in proactive personality, life satisfaction serves as an important resource for maintaining vigour at work. However, for individuals high in proactive personality, vigour levels remain relatively high regardless of life satisfaction, suggesting that their proactive disposition enables them to generate their own energetic resources independent of general life satisfaction.

### Moderation analysis 2: dedication as outcome

The overall moderation model was significant, *F*(4,96) = 11.52, *p* < 0.001, R^2^ = 0.32, accounting for 32% of the variance in Dedication. However, the interaction term (SWL × PPS) was not statistically significant (β = −1.04, SE = 0.63, t = −1.65, *p* = 0.10, 95% CI [−2.29, 0.21]). This indicates that proactive personality does not significantly moderate the relationship between life satisfaction and Dedication. Therefore, H4b was not supported.

### Moderation analysis 3: absorption as outcome

The overall moderation model was significant, *F*(4,96) = 17.63, *p* < 0.001, R^2^ = 0.42, accounting for 42% of the variance in Absorption. However, the interaction term (SWL × PPS) was not statistically significant (β = −0.89, SE = 0.47, t = −1.90, *p* = 0.06, 95% CI [−1.82, 0.04]). Although the *p*-value approached significance, it did not reach the conventional 0.05 threshold. Therefore, H4c was not supported.

### Summary of hypothesis testing

Table 4 summarises the support for each hypothesis.

**Table 4:** Summary of hypothesis testing results

Hypothesis	Description	Result
H1a	Life satisfaction → Vigour (+)	Supported
H1b	Life satisfaction → Dedication (+)	Not supported
H1c	Life satisfaction → Absorption (+)	Supported
H2a	Perceived stress → Vigour (-)	Supported
H2b	Perceived stress → Dedication (-)	Supported
H2c	Perceived stress → Absorption (-)	Not supported
H3a	Proactive personality → Vigour (+)	Supported
H3b	Proactive personality → Dedication (+)	Supported
H3c	Proactive personality → Absorption (+)	Supported
H4a	PPS moderates SWL → Vigour	Supported
H4b	PPS moderates SWL → Dedication	Not supported
H4c	PPS moderates SWL → Absorption	Not supported

In summary, proactive personality emerged as a consistent predictor of all three work engagement dimensions. Life satisfaction predicted Vigour and Absorption but not Dedication, whilst perceived stress predicted Vigour and Dedication but not Absorption. Most notably, proactive personality significantly moderated the relationship between life satisfaction and Vigour, such that life satisfaction was most important for individuals low in proactive personality. This moderation effect was not observed for Dedication or Absorption.

## Discussion

This study aimed to examine the relationships between satisfaction with life (SWL), perceived stress (PS), and proactive personality (PPS) with work engagement in teachers and support staff in an alternative education setting. Specifically, we tested whether these individual-level personal resources predicted the three dimensions of work engagement (Vigour, Dedication, and Absorption), and critically, whether proactive personality moderated the relationship between life satisfaction and engagement dimensions. Our findings provide three key contributions to the work engagement literature. First, we demonstrate that previously documented relationships between personal resources and work engagement extend to the severely understudied AP context, where unique challenges (high staff turnover, vulnerable student populations, limited resources) create a distinctive professional environment. Second, and most importantly from a theoretical perspective, we identified a significant moderation effect whereby proactive personality conditionally shaped the impact of life satisfaction on Vigour—a finding that advances Conservation of Resources theory [34, 74, 75] by demonstrating that stable personality traits influence how individuals mobilise their well-being resources for work investment. Third, we observed differential patterns across engagement dimensions: life satisfaction predicted Vigour and Absorption but not Dedication, whereas perceived stress predicted Vigour and Dedication but not absorption. This pattern suggests that engagement components may rely on distinct personal resource configurations.

Our most theoretically significant finding concerns the moderating role of proactive personality in the life satisfaction–Vigour relationship (H4a). As hypothesised, proactive personality significantly moderated this relationship, such that life satisfaction strongly predicted Vigour among individuals low in proactive personality but not for those high in proactive personality. This pattern supports a compensatory resources model, whereby highly proactive individuals—being inherently driven to influence their environment and seek opportunities for growth [65, 66], appear able to generate their own energetic resources or find engagement through active problem-solving irrespective of their baseline life satisfaction [12]. Their proactive disposition therefore serves as the primary engine of Vigour. Conversely, for individuals with lower proactive tendencies, overall life satisfaction acts as a vital personal resource, providing the psychological capital necessary to sustain energetic engagement in demanding AP roles [76, 77]. This finding extends both the JD-R framework and Conservation of Resources theory by demonstrating that personal resources do not operate uniformly across individuals—rather, stable personality characteristics determine which resources are most consequential for engagement. From a practical perspective, this suggests that well-being interventions designed to enhance life satisfaction may be particularly effective for educators who are less naturally proactive, while interventions for highly proactive individuals might more usefully focus on providing opportunities for environmental shaping and job crafting.

Importantly, this moderation effect was specific to Vigour and did not extend to Dedication (H4b) or Absorption (H4c), despite our hypotheses predicting otherwise. This specificity is theoretically informative, suggesting that the three engagement dimensions may vary in their sensitivity to personal resource configurations. Vigour, representing the energetic component of engagement, may be particularly dependent on general well-being (for less proactive individuals) or self-generated resources through proactive behaviour (for highly proactive individuals). In contrast, Dedication and Absorption appear to benefit additively from both life satisfaction and proactive personality, without interactive effects, suggesting these dimensions may draw upon different psychological processes or require different resource combinations.

Regarding the main effects (H1-H3), our findings both replicate and extend previous research while revealing important contextual nuances. Examining the relationship between life satisfaction and work engagement, our results partially supported the hypotheses and previous findings, indicating a positive relationship between these constructs. Specifically, life satisfaction positively predicted Vigour (H1a supported) and Absorption (H1c supported) but not Dedication (H1b not supported), thereby supporting the assertion that life satisfaction can act as an antecedent of work engagement [28]. Similar positive associations between subjective well-being and work engagement have been reported in educational contexts, including UK staff [78] and European professionals [79]. The present study advances understanding of these associations within a new educational context and professional population. It specifically examines this relationship within an AP setting, highlighting differential effects across engagement dimensions. The finding that life satisfaction predicts Vigour and Absorption but not Dedication is novel and suggests that general well-being may be more critical for sustaining energy and immersion in work than for fostering emotional commitment to one's role. This pattern may reflect the unique context of AP, where educators' dedication is likely driven by vocational commitment to supporting vulnerable students than by general life satisfaction—a possibility warranting further investigation.

This relationship is potentially highly significant for AP settings, as it suggests that efforts to enhance educators' overall life satisfaction may directly foster increases in specific dimensions of work engagement (particularly Vigour), rather than assuming a unidirectional influence from work engagement to improved life satisfaction. Although studies conducted directly in AP are scarce, qualitative and policy research in these contexts (Dunnett et al., 2025), [9] highlight the relational, emotional, and structural demands placed on staff. These demands suggest that staff well-being and satisfaction are likely foundational to their capacity to engage fully at work. Furthermore, broader literature in mainstream education indicates that teacher well-being interventions reduce burnout, enhance emotional regulation, and contribute to improved student outcomes (Buonomo et al., 2022, Carroll et al., 2021). Given that AP settings often involve students with additional challenges, the extension of life satisfaction’s influence on engagement may be particularly impactful in this context. Therefore, interventions aimed at improving educators’ life satisfaction may yield positive outcomes for both staff well-being and student engagement within alternative education, especially for those less proactive in shaping their work environments.

It is important to note that life satisfaction judgements are shaped by individual experiences and encompass multiple life domains, reflecting personal values [80, 81]. This multifaceted nature aligns with the evolved Job Demands-Resources (JD-R) theory, which emphasises the dynamic role of personal resources in influencing work environments and sustaining engagement [82, 83]. Our findings extend JD-R theory by revealing that personal resources interact with personality traits to influence engagement, rather than operating solely through additive main effects. Given the intricate interplay between life satisfaction and work engagement, particularly within alternative education settings, future research should aim to clarify which specific components of life satisfaction most strongly impact engagement and how other personal resources modulate this effect.

Regarding proactive personality, our results showed a consistent positive relationship with all three work engagement subscales (Vigour, Dedication, and Absorption) (H3a, H3b, and H3c all supported), with standardised regression coefficients indicating robust effects. This finding supports previous research linking proactive personality to work engagement in educational contexts [63, 64] and confirms that this relationship extends to the alternative provision setting. However, our study advances beyond simple replication by demonstrating that proactive personality serves multiple functions: it directly predicts all engagement dimensions and additionally moderates the life satisfaction–Vigour relationship. This dual role—as both a direct predictor and a conditional moderator—positions proactive personality as a particularly critical individual difference in alternative provision contexts. From a JD-R perspective, proactive personality appears to function as a meta-resource that both generates engagement directly and determines how other personal resources (such as life satisfaction) are mobilised for work investment [26].

The particularly strong effect of proactive personality on Vigour compared to Dedication and Absorption may reflect the nature of AP work, where educators must continually adapt to unpredictable student needs, navigate resource constraints, and create solutions in the absence of systemic support [1, 23, 30, 36]. Proactive individuals' inherent drive to influence their environment [65] may be particularly valuable for sustaining the energy (Vigour) required in this context. This interpretation aligns with JD-R theory [21], suggesting that individuals with proactive personalities are more inclined to actively shape their work environment, leading to enhanced engagement [84]. Previous research has demonstrated that proactive personality enables individuals to initiate change by drawing upon their own resources, thereby increasing their motivation and engagement in their work [57],Van Wingerden, 2017). Furthermore, proactivity is associated with increased work immersion and engagement in new teachers, particularly in early career stages [62, 63]. Our findings extend this research by evidencing these effects in alternative provision and by revealing proactive personality's moderating function. The robust direct effect indicates that fostering proactive behaviour among staff could be a valuable strategy for enhancing work engagement in alternative settings, though organisations must balance this with concerns about resource depletion from excessive proactivity.

While research on proactive personality's impact on work engagement in educational settings is developing, our findings align with evidence indicating that both personal and job resources are crucial for engagement. Previous research has shown that proactive strategies enable educators to utilise personal resources more effectively, potentially reducing perceived stress and enhancing coping strategies and engagement [32], and that teachers with proactive personalities demonstrate higher self-efficacy and work engagement, indicating a greater likelihood of developing job resources [63]. However, our results introduce a crucial caveat: proactivity appears most beneficial when it enables individuals to compensate for limitations in other resources (such as lower life satisfaction). This suggests that educational institutions should carefully balance supporting proactive staff with job crafting opportunities while monitoring resource investment to optimise work engagement and its consequent benefits for both educators and students, as excessive proactivity may lead to decreased work engagement due to overinvestment of personal resources [85, 86]. Regarding perceived stress, our results partially supported hypotheses, as perceived stress negatively predicted both Vigour (H2a supported) and Dedication (H2b supported), but not Absorption (H2c not supported). This differential pattern is theoretically informative, suggesting that perceived stress depletes the energetic (Vigour) and emotionally committed (Dedication) aspects of engagement while leaving the cognitive state of immersion (Absorption) unaffected. One interpretation is that alternative provision educators may experience a dissociative pattern whereby stress depletes their energy and commitment but does not prevent them from becoming absorbed in their work when actively engaged with students—possibly because the intrinsically rewarding nature of the work itself provides a form of respite or flow experience that is somewhat insulated from general stress perceptions [87]. Alternatively, this pattern may reflect measurement issues.

Importantly, however, coping strategies were not measured in this study, representing a key limitation. While our data reveal associations between perceived stress and specific engagement dimensions, we cannot identify the mechanisms through which some individuals maintain engagement despite stress, nor determine which coping strategies might be most effective. Previous research suggests that stress mindset [46] and coping resources [50, 54] significantly influence whether stress depletes or enhances engagement, but our study could not directly test these mechanisms. This interpretation aligns with recent research by Zhang et al. [55] and Misu [18], which found high work engagement despite substantial stressors, suggesting a more complex relationship between stress and engagement than simple depletion models would predict. Research linking perceived stress to personality and mindset demonstrates that individual differences in stress perception and coping abilities can significantly influence work engagement [46, 50]. The present findings align with the idea that stress is shaped by personal mindset, with individuals interpreting it as either enhancing or debilitating [47]. Our findings therefore suggest a complex stress–engagement relationship within alternative provision settings but cannot definitively characterise this complexity without measures of coping strategies and stress mindset. This represents an important avenue for future research. The generic nature of the stress scale, does not account for individual perceptions and specific life contexts [49], may fail to capture the intricate realities of stress in this specialised educational environment. Consequently, future research should prioritise the development of bespoke stress measures for alternative education, alongside a deeper exploration of the role that coping strategies play in sustaining engagement amidst stress. Such an approach would not only provide more reliable insights but also inform the development of targeted supportive strategies, potentially leading to improved well-being and engagement among staff in these challenging educational contexts.

### Theoretical contributions

This study makes three significant theoretical contributions to the work engagement literature, particularly within the JD-R framework. First, and most importantly, we identified a significant interaction between personal resources whereby proactive personality moderated the life satisfaction–Vigour relationship. This finding advances Conservation of Resources theory [34] and the JD-R framework [12] by demonstrating that personal resources do not operate uniformly or additively—rather, stable personality traits conditionally determine how other resources (such as life satisfaction) are mobilised for work investment. This challenges the implicit assumption in JD-R research that personal resources simply accumulate to predict engagement, revealing instead a more dynamic and interactive resource structure. Recent developments in JD‑R theory highlight that personal resources can display nonlinear and compensatory effects, where their benefits depend on contextual and individual conditions rather than additive accumulation [88, 89]. The observed pattern—where life satisfaction predicts Vigour for low‑proactive individuals but not for highly proactive individuals—supports a compensatory resources perspective: when one resource is limited (e.g., low proactive personality), others (e.g., life satisfaction) become more critical, whereas when key resources are abundant, additional resources offer diminishing returns [90, 91]. This principle accords with the emerging JD‑R 3.0 framework, which conceptualises engagement as the outcome of complex, conditional interactions among overlapping personal and job resources rather than simple linear effects [92].

Second, our findings revealed differential patterns of prediction across the three engagement dimensions—Vigour, Dedication, and Absorption—indicating that these facets may depend on distinct combinations of personal resources. Specifically, life satisfaction predicted Vigour and Absorption but not Dedication, while perceived stress predicted Vigour and Dedication but not Absorption. This pattern aligns with recent evidence indicating that the dimensions of engagement operate differently and may be linked to divergent motivational or affective mechanisms [93]. Conceptualising work engagement as a single, unified construct may obscure important differences across its underlying dimensions [94]. By differentiating among the three components, our findings support arguments that distinct predictors and resource pathways underpin Vigour, Dedication, and Absorption, and that targeted interventions should cultivate specific personal resources aligned with each dimension—for instance, emotional regulation strategies may enhance Vigour, whereas meaning‑oriented resources may strengthen Dedication [83, 87].

Third, we extend work engagement research into the severely understudied AP context, demonstrating that relationships established in mainstream educational settings largely replicate within this unique professional environment while also revealing context-specific patterns. For example, the non-significance of life satisfaction for Dedication, may reflect the vocational commitment characteristic of educators working with vulnerable students.

### Practical contributions

From a practical perspective, our findings hold important implications for supporting educator well-being and engagement in alternative provision settings. The moderation effect indicates that well-being interventions should be tailored to individual differences. Initiatives designed to enhance general life satisfaction (e.g., work-life balance programmes, general well-being support) may be particularly effective for educators who are less naturally proactive, whilst interventions for highly proactive individuals might more usefully focus on providing opportunities for job crafting, autonomy, and environmental shaping. This personalised approach to intervention design marks a departure from one-size-fits-all well-being programmes. Additionally, the strong direct effects of proactive personality suggest that recruitment and selection processes could consider proactive tendencies as relevant individual differences, though organisations must balance this with awareness of potential resource depletion from excessive proactivity. Finally, the differential effects across engagement dimensions suggest that organisations should monitor staff well-being and engagement using measures that distinguish between Vigour, Dedication, and Absorption, as interventions may need to target specific dimensions experiencing the greatest depletion [8], Dunnett et al., 2025).

A key strength is the inclusion of support staff, often overlooked in educational research despite their integral role [95, 96]. Our results provide valuable insights into the personal factors that affect work engagement, highlighting how psychological availability impacts engagement and the significance of personal resources within the JD-R model [21, 82]. However, our most important contribution lies in demonstrating that personal resources interact rather than simply accumulate—a finding with implications for both theory and practice in work engagement research.

### Limitations and future directions

However, several limitations must be acknowledged. The cross-sectional design limits causal inferences, particularly regarding the directionality of relationships. Whilst we tested life satisfaction as a predictor of work engagement based on theoretical rationale, the reverse relationship (engagement predicting life satisfaction) or bidirectional relationships remain possible and cannot be ruled out with our data. This is particularly relevant given longitudinal evidence of bidirectional relationships between life satisfaction and engagement over time [42]. Future research employing longitudinal or experimental designs is essential to establish temporal precedence and causal mechanisms, particularly regarding how work engagement may fluctuate over time [19, 57], how the moderation effect might change as individuals develop in their careers, and whether interventions targeting life satisfaction causally increase engagement for low-proactive individuals as our cross-sectional findings suggest. The lack of specificity in the SWL and PS measures hinders a deeper understanding of the factors influencing life satisfaction and stress perceptions [37, 49]. Additionally, scales such as the Teacher Stress Inventory [97], or subscales from comprehensive occupational stress questionnaires focusing on workload, pupil behaviour, and emotional demands (e.g., [98, 99]), may be more suitable than a generic stress measure. These specific instruments are inherently designed to resonate with the professional experiences of educators, which could enable the capture of relevant stressors with greater precision and lead to more robust reliability in future data. We did not measure coping strategies, stress mindset, or job crafting behaviours—variables that theory suggests would be crucial for understanding the mechanisms through which proactive personality and stress influence engagement. Future research must incorporate these mediating variables to fully test the theoretical processes we propose.

Beyond addressing these measurement and design limitations, future research could build upon these findings by exploring the precise mechanisms through which life satisfaction and proactive personality interact to influence work engagement. Specifically, studies should test whether the moderation effect operates through job crafting behaviours (i.e., whether highly proactive individuals engage in more job crafting, which compensates for lower life satisfaction) or through cognitive-motivational processes (i.e., whether proactive individuals simply require less external resource input due to their self-generated motivation). Additionally, research should examine whether similar moderation effects occur with other personal resources (e.g., self-efficacy, optimism) or whether the pattern is specific to life satisfaction. Longitudinal and mixed-methods approaches are crucial to uncover potential bidirectional relationships between variables, such as work engagement as both a consequence and antecedent of proactive behaviour [62], and its dynamic relationship with life satisfaction [28]. Applying the JD-R theory to investigate specific job demands and resources impacting work engagement in educational settings could yield valuable insights [12, 21]. Studies on leadership's role in facilitating job crafting and work engagement, particularly in the context of educational policy constraints, would be beneficial [30, 60]. Developing more context-specific measures for stress in alternative education settings is also imperative, given the complexity of stress perception in this environment and the identified reliability issues with generic scales [49]. Finally, intervention research testing whether tailored well-being programmes (targeting life satisfaction for low-proactive individuals and job crafting opportunities for high-proactive individuals) causally enhance engagement would provide crucial evidence for translating our findings into practice.

## Conclusion

In conclusion, this study advances work engagement theory by emphasising that individual-level personal resources interact conditionally rather than simply accumulating additively. Our key finding—that proactive personality moderates the life satisfaction–Vigour relationship—demonstrates that the impact of one personal resource (life satisfaction) on the energetic component of engagement (Vigour) depends critically upon another personal resource (proactive personality). This has important implications for both theory and practice: theoretically, it suggests that JD-R models should incorporate resource interactions alongside main effects; practically, it suggests that well-being interventions should be tailored to individual differences, with life satisfaction enhancement particularly beneficial for less proactive educators. Given the demanding nature of teaching in alternative provision and ongoing retention concerns [51, 100, 101], recognising and nurturing these personal resources and understanding their interactions is paramount. Whilst proactive personality consistently predicted all engagement dimensions and demonstrated the hypothesised moderation effect for Vigour, our findings also reveal dimension-specific patterns (e.g., stress affecting Vigour and Dedication but not Absorption) that warrant further investigation. Prioritising the development of personal resources that foster both well-being and engagement at work is imperative for schools and alternative education settings, not only for staff well-being but also for ensuring positive student outcomes. While perceived stress demonstrated a more nuanced relationship with engagement in this study, cultivating an environment that promotes effective coping strategies remains crucial [53]. Ultimately, a deeper, context-specific understanding of how these personal resources interact will enable researchers and practitioners to develop targeted strategies for creating more resilient, engaged, and effective educational environments that benefit both staff and students alike.

## Data Availability

The data that support the findings of this study are available upon request from the corresponding author. The data are not publicly available due to privacy or ethical restrictions.

## References

[1] Malcolm A. Sustaining post-16 destinations from alternative provision: a review of the data and the perspectives of heads from low, mid and high performing schools. Emot Behav Diffic. 2022;27(1):20–42. 10.1080/13632752.2022.2025646.

[2] Department for Education. (2023). School workforce in England: Reporting year 2023. Department for Education.https://explore-education-statistics.service.gov.uk/find-statistics/school-workforce-in-england/2023

[3] Taylor AM, McCluskey G. “Alternative” education provision: a mapping and critique. Oxf Rev Educ. 2024;50(4):512–30. 10.1080/03054985.2024.2373067.

[4] Klassen RM, Yerdelen S, Durksen TL. Measuring teacher engagement: development of the engaged teachers scale (ETS). Front Learn Res. 2013;1(2):33–52. 10.14786/flr.v1i2.44.

[5] Department for Education. (2024). School workforce in England: November 2024 . UK Government.https://explore-education-statistics.service.gov.uk/find-statistics/school-workforce-in-england

[6] IntegratED Annual Report (2023). State of the Nation: School Exclusion and Alternative Provision in England. IntegratED Partnership. https://www.integrated.org.uk/wp-content/uploads/2023/12/AnnualReport23_v6-002-wecompress.com_.pdf

[7] Power S. Outsourcing trouble: a home international comparison of alternative provision. Br J Educ Stud. 2025;73(1):1–23. 10.1080/00071005.2025.2481869. 10.1080/00071005.2025.2481869 PMC12327216 40772254

[8] Gibson S, Wilson R, Kingsford-Smith A. Teacher wellbeing in alternative provision: predictors and outcomes. Br J Educ Stud. 2022;70(3):387–405.

[9] Ofsted. (2022). Annual report and accounts: Education, children’s services and skills 2021/22. Office for Standards in Education, Children's Services and Skills. https://www.gov.uk/government/publications/ofsted-annual-report-202223-education-childrens-services-and-skills

[10] Mäkinen M. Becoming engaged in inclusive practices: narrative reflections on teaching as descriptors of teachers’ work engagement. Teach Teach Educ. 2013;35:51–61. 10.1016/j.tate.2013.05.005.

[11] Bakker AB, Albrecht SL, Leiter MP. Key questions regarding work engagement. Eur J Work Organ Psychol. 2011;20(1):4–28. 10.1080/1359432X.2010.485352.

[12] Bakker AB, Demerouti E. Job demands–resources theory: taking stock and looking forward. J Occup Health Psychol. 2017;22(3):273. 10.1037/ocp0000056. 27732008 10.1037/ocp0000056

[13] Christian MS, Garza AS, Slaughter JE. Work engagement: a quantitative review and test of its relations with task and contextual performance. Personnel Psychol. 2011;64(1):89–136. 10.1111/j.1744-6570.2010.01203.x.

[14] Kahn WA. Psychological conditions of personal engagement and disengagement at work. Acad Manag J. 1990;33(4):692–724. 10.2307/256287.

[15] Hakanen JJ, Bakker AB, Schaufeli WB. Burnout and work engagement among teachers. J Sch Psychol. 2006;43(6):495–513. 10.1016/j.jsp.2005.11.001.

[16] Schaufeli WB, Salanova M, González-Romá V, Bakker AB. The measurement of engagement and burnout: a two-sample confirmatory factor analytic approach. J Happiness Stud. 2002;3(1):71–92. 10.1023/A:1015630930326.

[17] Xanthopoulou D, Bakker AB, Demerouti E, Schaufeli WB. Work engagement and financial returns: a diary study on the role of job and personal resources. J Occup Organ Psychol. 2009;82(1):183–200. 10.1348/096317908X285633.

[18] Misu SI. Teacher’s work engagement-change and adaptation during COVID-19 pandemic. Business Excellence and Management. 2020;10(SI 1):243–55. 10.24818/beman/2020.S.I.1-19.

[19] Bakker AB. Job demands-resources theory: ten years later. Annu Rev Organ Psychol Organ Behav. 2023. 10.1146/annurev-orgpsych-120920-053933.

[20] Ma Y. Boosting teacher work engagement: the mediating role of psychological capital through emotion regulation. Front Psychol. 2023;14:1240943. 10.3389/fpsyg.2023.1240943. 37720646 10.3389/fpsyg.2023.1240943 PMC10501726

[21] Demerouti E, Bakker AB, Nachreiner F, Schaufeli WB. The job demands-resources model of burnout. J Appl Psychol. 2001;86(3):499. 10.1037/0021-9010.86.3.499. 11419809

[22] Van Wingerden J, Bakker AB, Derks D. The longitudinal impact of a job crafting intervention. Eur J Work Organ Psychol. 2017;26(1):107–19. 10.1080/1359432X.2016.1224233.

[23] Malcolm A. Exclusions and alternative provision: piecing together the picture. Emot Behav Diffic. 2018;23(1):69–80. 10.1080/13632752.2017.1366099.

[24] Tadić M, Bakker AB, Oerlemans WG. Work happiness among teachers: a day reconstruction study on the role of self-concordance. J Sch Psychol. 2013;51(6):735–50. 10.1016/j.jsp.2013.07.002. 24295146 10.1016/j.jsp.2013.07.002

[25] Aldrup K, Carstensen B, Klusmann U. The role of teachers’ emotion regulation in teaching effectiveness: a meta-analytic review. Educ Psychol. 2024;59(1):1–21. 10.1080/00461520.2023.2282446.

[26] Sahli Lozano C, Wicki M, Wüthrich S, Setz F. A systematic review and meta-analysis of collective teacher efficacy’s relationships with outcomes in the job demands-resources model. Teach Teach Educ. 2025;159:105006. 10.1016/j.tate.2024.105006.

[27] Ruttledge R. A whole school approach to building relationships, promoting positive behaviour and reducing teacher stress in a secondary school. Educ Psychol Pract. 2022;38(3):237–58. 10.1080/02667363.2022.2070456.

[28] Ferreira P, Gabriel C, Faria S, Rodrigues P, Sousa Pereira M. What if employees brought their life to work? The relation of life satisfaction and work engagement. Sustainability. 2020;12(7):2743. 10.3390/su12072743.

[29] Topchyan R, Woehler C. Do teacher status, gender, and years of teaching experience impact job satisfaction and work engagement? Educ Urban Soc. 2021;53(2):119–45. 10.1177/0013124520926161.

[30] Ofsted. (2024). Alternative provision in local areas in England: A thematic review.https://www.gov.uk/government/publications/alternative-provision-in-local-areas-in-england-a-thematic-review

[31] Owen C, Woods K, Stewart A. A systematic literature review exploring the facilitators and barriers of reintegration to secondary mainstream schools through ‘alternative provision.’ Emotional and Behavioural Difficulties. 2021;26(3):322–38. 10.1080/13632752.2021.1963119.

[32] De Stasio S, Fiorilli C, Benevene P, Boldrini F, Ragni B, Pepe A, et al. Subjective happiness and compassion are enough to increase teachers’ work engagement? Front Psychol. 2019;10:2268. 10.3389/fpsyg.2019.02268. 31681081 10.3389/fpsyg.2019.02268 PMC6811656

[33] Field LK, Buitendach JH. Happiness, work engagement and organisational commitment of support staff at a tertiary education institution in South Africa. SA J Ind Psychol. 2011;37(1):01–10. 10.4102/sajip.v37i1.946.

[34] Hobfoll SE. Conservation of resources: a new attempt at conceptualizing stress. Am Psychol. 1989;44(3):513. 10.1037/0003-066X.44.3.513. 2648906 10.1037//0003-066x.44.3.513

[35] Fredrickson BL. The role of positive emotions in positive psychology: the broaden-and-build theory of positive emotions. Am Psychol. 2001;56(3):218–26. 10.1037/0003-066X.56.3.218. 11315248 10.1037//0003-066x.56.3.218 PMC3122271

[36] Ofsted. (2019). Summary and recommendations: teacher well-being research report.https://www.gov.uk/government/publications/teacher-well-being-at-work-in-schools-and-further-education-providers/summary-and-recommendations-teacher-well-being-research-report

[37] Diener ED, Emmons RA, Larsen RJ, Griffin S. The satisfaction with life scale. J Pers Assess. 1985;49(1):71–5. 10.1207/s15327752jpa4901_13. 16367493 10.1207/s15327752jpa4901_13

[38] Pavot W, Diener E. Review of the satisfaction with life scale. Psychol Assess. 1993;5(2):164–72. 10.1037/1040-3590.5.2.164.

[39] Warr P, Nielsen K. Wellbeing and work performance. In: Diener E, Oishi S, Tay L, editors. Handbook of well-being. DEF Publishers; 2018. p. 377–89.

[40] Çelik E. Examining the mediating effect of subjective vitality in the proactive personality and life satisfaction relationship. Int J Happiness Dev. 2017;3(4):289–302. 10.1504/ijhd.2017.10008779.

[41] Eldor L, Harpaz I, Westman M. Life satisfaction as a predictor of work engagement: a resource perspective. J Vocat Behav. 2020;122:103475. 10.1016/j.jvb.2020.103475.

[42] Upadyaya K, Salmela-Aro K. Developmental dynamics between young adults’ life satisfaction and engagement with studies and work. Longitud Life Course Stud. 2017;8(1):20–34. 10.14301/llcs.v8i1.398.

[43] Barnett BR, Bradley L. The impact of organisational support for career development on career satisfaction. Career Dev Int. 2007;12(7):617–36. 10.1108/13620430710834396.

[44] Žnidaršič J, Marič M. Relationships between work-family balance, job satisfaction, life satisfaction and work engagement among higher education lecturers. Organizacija. 2021;54(3):227–37. 10.2478/orga-2021-0015.

[45] Thomson P, Pennacchia J. Hugs and behaviour points: alternative education and the regulation of “excluded” youth. Int J Inclusive Educ. 2016;20(6):622–40. 10.1080/13603116.2015.1102340.

[46] Casper A, Sonnentag S, Tremmel S. Mindset matters: the role of employees’ stress mindset for day-specific reactions to workload anticipation. Eur J Work Organ Psychol. 2017;26(6):798–810. 10.1080/1359432X.2017.1374947.

[47] Keech JJ, Cole KL, Hagger MS, Hamilton K. The association between stress mindset and physical and psychological wellbeing: testing a stress beliefs model in police officers. Psychol Health. 2020;35(11):1306–25. 10.1080/08870446.2020.1743841. 32212946 10.1080/08870446.2020.1743841

[48] Keech JJ, Hagger MS, O’Callaghan FV, Hamilton K. The influence of university students’ stress mindsets on health and performance outcomes. Ann Behav Med. 2018;52(12):1046–59. 10.1093/abm/kay008. 30418523 10.1093/abm/kay008

[49] Cohen, S., Kamarck, T., & Mermelstein, R. (1994). Perceived stress scale. In S. Cohen, T. Kamarck, & R. Mermelstein (Eds.), *Measuring stress: A guide for health and social scientists* (Vol. 10, pp. 1–2). Oxford University Press.

[50] Carver CS, Connor-Smith J. Personality and coping. Annu Rev Psychol. 2010;61:679–704. 10.1146/annurev.psych.093008.100352. 19572784 10.1146/annurev.psych.093008.100352

[51] Van Wingerden J, Poell RF. Meaningful work and resilience among teachers: the mediating role of work engagement and job crafting. PLoS One. 2019;14(9):e0222518. 10.1371/journal.pone.0269347. 31536544 10.1371/journal.pone.0222518 PMC6752839

[52] Miranda AR, Rivadero L, Bruerad JÁ, Villarreal V, Bernioa LY, de los Ángeles Baydas L, et al. Examining the relationship between engagement and perceived stress-related cognitive complaints in the Argentinian working population. Eur J Psychol. 2020;16(1):12. 10.5964/ejop.v16i1.1832. 33680167 10.5964/ejop.v16i1.1832 PMC7913026

[53] Richards J. Teacher stress and coping strategies: a national snapshot. Educ Forum. 2012;76(3):299–316. 10.1080/00131725.2012.682837.

[54] Roeser RW, Schonert-Reichl KA, Jha A, Cullen M, Wallace L, Wilensky R, et al. Mindfulness training and reductions in teacher stress and burnout: results from two randomized, waitlist-control field trials. J Educ Psychol. 2013;105(3):787. 10.1037/a0032093.

[55] Zhang M, Zhang P, Liu Y, Wang H, Hu K, Du M. Influence of perceived stress and workload on work engagement in front-line nurses during COVID-19 pandemic. J Clin Nurs. 2021;30(11–12):1584–95. 10.1111/jocn.15707. 33590524 10.1111/jocn.15707 PMC8014711

[56] Bateman TS, Crant JM. The proactive component of organizational behavior: a measure and correlates. J Organ Behav. 1993;14(2):103–18. 10.1002/job.4030140202.

[57] Bakker AB, Tims M, Derks D. Proactive personality and job performance: the role of job crafting and work engagement. Hum Relat. 2012;65(10):1359–78. 10.1177/0018726712453471.

[58] Claes R, Van Loo K. Relationships of proactive behaviour with job-related affective well-being and anticipated retirement age: an exploration among older employees in Belgium. Eur J Ageing. 2011;8(4):233–41. 10.1007/s10433-011-0203-7. 28798653 10.1007/s10433-011-0203-7 PMC5547361

[59] Grant AM, Ashford SJ. The dynamics of proactivity at work. Annu Rev Organ Psychol Organ Behav. 2020;7:249–76. 10.1146/annurev-orgpsych-012119-044850.

[60] Tornau K, Frese M. Construct clean-up in proactivity research: a meta-analysis on the nomological net of work-related proactivity concepts and their incremental validities. Appl Psychol. 2013;62(1):44–96. 10.1111/j.1464-0597.2012.00514.x.

[61] Caniëls MC, Semeijn JH, Renders IH. Mind the mindset! The interaction of proactive personality, transformational leadership and growth mindset for engagement at work. Career Dev Int. 2018;23(3):291–309. 10.1108/CDI-11-2016-0194.

[62] Cooper-Thomas HD, Paterson NL, Stadler MJ, Saks AM. The relative importance of proactive behaviors and outcomes for predicting newcomer learning, well-being, and work engagement. J Vocat Behav. 2014;84(3):318–31. 10.1016/j.jvb.2014.02.007.

[63] Li M, Wang Z, Gao J, You X. Proactive personality and job satisfaction: the mediating effects of self-efficacy and work engagement in teachers. Curr Psychol. 2017;36(1):48–55. 10.1007/s12144-015-9383-1.

[64] Jorg F. Teachers’ proactive behaviour and job design: a multilevel perspective. Br J Educ Psychol. 2024;94(2):215–31. 10.1111/bjep.12642. 10.1111/bjep.12642 37957784

[65] Crant JM. Proactive personality and work success revisited. J Appl Psychol. 2021;106(4):585–600. 10.1037/apl0000878.

[66] Parker SK, Bindl UK, Strauss K. Making things happen: a model of proactive motivation. J Manage. 2010;36(4):827–56. 10.1177/0149206310363732.

[67] Faul F, Erdfelder E, Lang A-G, Buchner A. G*power 3: a flexible statistical power analysis program for the social, behavioral, and biomedical sciences. Behav Res Methods. 2007;39:175–91. 10.3758/BF03193146. 17695343 10.3758/bf03193146

[68] Information Commissioner's Office. (2021). Guide to the UK General Data Protection Regulation (UK GDPR): Right of access. https://ico.org.uk/for-organisations/guide-to-data-protection/guide-to-the-uk-gdpr/individual-rights/right-of-access/

[69] Cohen S, Kamarck T, Mermelstein R. A global measure of perceived stress. J Health Soc Behav. 1983. 10.2307/2136404. 6668417

[70] Cohen S, Janicki‐Deverts D. Who’s stressed? Distributions of psychological stress in the United States in probability samples from 1983, 2006, and 2009 1. J Appl Soc Psychol. 2012;42(6):1320–34. 10.1111/j.1559-1816.2012.00900.x.

[71] Collie RJ, Shapka JD, Perry NE. School climate and social–emotional learning: predicting teacher stress, job satisfaction, and teaching efficacy. J Educ Psychol. 2012;104(4):1189–204. 10.1037/a0029356.

[72] Granziera H, Perera HN, McIlveen P. Teacher well-being: a systematic review of the research literature from 2012 to 2020. Educ Res Rev. 2021;33:100386. 10.1016/j.edurev.2021.100386.

[73] Hayes, A. F. (2012). PROCESS: a versatile computational tool for observed variable mediation, moderation and conditional process modeling [White paper]. Available online at:

[74] Fröhlich P, Radaca E, Diestel S. When happiness strengthens engagement and performance: the role of happiness at work as a resource for experienced employees and newcomers. Front Psychol. 2025;16:1560010. 10.3389/fpsyg.2025.1560010. 40799338 10.3389/fpsyg.2025.1560010 PMC12341457

[75] Wang, Y., & Liu, X. (2025). Wise proactivity: Antecedents, outcomes, and a mechanism. *Journal of Organizational Behavior*. Advance online publication. 10.1002/job.2887

[76] Delhey J, Hess S, Boehnke K, Deutsch F, Eichhorn J, Kühnen U, et al. Life satisfaction during the COVID-19 pandemic: the role of human, economic, social, and psychological capital. J Happiness Stud. 2023. 10.1007/s10902-023-00676-w.

[77] Gürbüz S, Schaufeli WB, Freese C, Brouwers EPM. Fueling creativity: HR practices, work engagement, personality, and autonomy. Int J Hum Resour Manage. 2024;35(22):3770–99. 10.1080/09585192.2024.2429125.

[78] Blanchflower DG, Bryson A. Life satisfaction in Western Europe and the gradual vanishing of the U-shape in age. Mental Health and Wellbeing. 2025;3(2):MHealthWellB7877. 10.20935/MHealthWellB7877.

[79] Wieczorek-Szymańska A. Work engagement and work–life balance across European Union countries. Management Papers. 2024;22(3):195–212.

[80] Diener E, Oishi S, Tay L. Advances in subjective well-being research. Nat Hum Behav. 2018;2(4):253–60. 10.1038/s41562-018-0307-6. 30936533 10.1038/s41562-018-0307-6

[81] Erdogan B, Bauer TN, Truxillo DM, Mansfield LR. Whistle while you work: a review of the life satisfaction literature. J Manage. 2012;38(4):1038–83. 10.1177/0149206311429379.

[82] Bakker AB, Demerouti E. Job demands–resources theory: frequently asked questions. J Occup Health Psychol. 2024;29(3):188–200. 10.1037/ocp0000376. 38913705 10.1037/ocp0000376

[83] Mazzetti G, Robledo E, Vignoli M, Topa G, Guglielmi D, Schaufeli WB. Work engagement: a meta-analysis using the job demands-resources model. Psychol Rep. 2023;126(3):1069–107. 10.1177/00332941211051988. 34886729 10.1177/00332941211051988

[84] Bakker AB, Demerouti E, Sanz-Vergel AI. Burnout and work engagement: the JD–R approach. Annu Rev Organ Psychol Organ Behav. 2014;1(1):389–411. 10.1146/annurev-orgpsych-031413-091235.

[85] Cangiano F, Bindl UK, Parker SK. The “hot” side of proactivity. In: Parker SK, Bindl UK, editors. Proactivity at work: Making things happen in organizations. Routledge; 2016. p. 355–84.

[86] Ouyang K, Cheng BH, Lam W, Parker SK. Enjoy your evening, be proactive tomorrow: how off-job experiences shape daily proactivity. J Appl Psychol. 2019;104(8):1003. 10.1037/apl0000391. 30730165 10.1037/apl0000391

[87] Muehlbacher F, Mejeh M, Keller MM, Hagenauer G. Teachers’ daily positive and negative affect and their relationship with teachers’ emotion regulation strategies and daily work engagement: results of a diary study among team teachers. Soc Psychol Educ. 2024;27:3369–98. 10.1007/s11218-024-09951-x.

[88] Ji T, de Jonge J, Peeters MCW, Taris TW. Matching job demands and job resources as linear and non-linear predictors of employee vigor and sustainable performance. Hum Perform. 2024;37(3):81–99. 10.1080/08959285.2024.2332683.

[89] Li Y, Chen C, Yuan Y. Evolving the job demands-resources framework to JD-R 3.0: the impact of after-hours connectivity and organizational support on employee psychological distress. Acta Psychol. 2025;253:104710. 10.1016/j.actpsy.2025.104710. 10.1016/j.actpsy.2025.104710 39799928

[90] Chen IS. Extending the job demands‑resources model to home demands and resources: testing the boost/buffer hypotheses for work engagement. Stress Health. 2024;40(1):131–47. 10.1002/smi.3362. 10.1002/smi.3362 38197865

[91] Van Zyl, L. E., Cornelisse, M. A., Le Blanc, P., & Rothmann, S. Sr. (2025). Cracks in the JD-R model? The failure of strengths use, job crafting, and home-work spillover to support wellbeing during COVID-19 [Perspective]. Front Psychol, 16:1532083. 10.3389/fpsyg.2025.1532083 10.3389/fpsyg.2025.1532083 PMC12199280 40575790

[92] Onyishi IE, Nohe C, Ugwu FO, Amazue LO, Hertel G. When high work engagement is negative for family tasks: mechanisms and boundary conditions. Front Psychol. 2024;15:1403701. 10.3389/fpsyg.2024.1403701. 38993350 10.3389/fpsyg.2024.1403701 PMC11238601

[93] Reig‑Botella A, Ramos‑Villagrasa PJ, del Fernández‑ Río E, Clemente M. Don’t curb your enthusiasm! The role of work engagement dimensions in predicting distinct performance outcomes. Journal of Work and Organizational Psychology. 2024;40(1):45–58. 10.5093/jwop2024a5.

[94] Demerouti E, Bakker AB. Job demands-resources theory in times of crises: new propositions. Organ Psychol Rev. 2022;13(3):209–36. 10.1177/20413866221135022.

[95] Jerrim, J. (2024). The link between school leadership, staff job satisfaction and retention. British Educational Research Journal. Advance online publication. 10.1002/berj.4134

[96] UNISON. (2024). School support staff survey 2024 [Report]. UNISON. https://www.unison.org.uk/content/uploads/2024/11/Stars-24-final-report_021224.pdf

[97] Fimian MJ. The development of an instrument to measure occupational stress in teachers: the Teacher Stress Inventory. J Occup Psychol. 1984;57(4):277–93. 10.1111/j.2044-8325.1984.tb00169.x.

[98] Cooper CL, Sloan SJ, Williams S. Occupational Stress Indicator: Management Guide. NFER-Nelson; 1988.

[99] Karasek RA. Job demands, job decision latitude, and mental strain: implications for job redesign. Adm Sci Q. 1979;24(2):285–308. 10.2307/2392498.

[100] Department for Education (2021, 9^th^ September). School Workforce in England. Reporting year 2020. GOV.UK.https://explore-education-statistics.service.gov.uk/find-statistics/school-workforce-in-england

[101] Ward, H (2019, 27^th^ June). One in three teachers leaves within five years. Times Educational Supplement.https://www.tes.com/news/one-three-teachers-leaves-within-five-years

